# *Mamld1* Knockdown Reduces Testosterone Production and *Cyp17a1* Expression in Mouse Leydig Tumor Cells

**DOI:** 10.1371/journal.pone.0019123

**Published:** 2011-04-29

**Authors:** Michiko Nakamura, Maki Fukami, Fumihiro Sugawa, Mami Miyado, Katsuya Nonomura, Tsutomu Ogata

**Affiliations:** 1 Department of Molecular Endocrinology, National Research Institute for Child Health and Development, Tokyo, Japan; 2 Department of Renal and Genitourinary Surgery, Hokkaido University Graduate School of Medicine, Sapporo, Japan; Texas A&M University, United States of America

## Abstract

**Background:**

*MAMLD1* is known to be a causative gene for hypospadias. Although previous studies have indicated that *MAMLD1* mutations result in hypospadias primarily because of compromised testosterone production around the critical period for fetal sex development, the underlying mechanism(s) remains to be clarified. Furthermore, although functional studies have indicated a transactivation function of *MAMLD1* for the non-canonical Notch target *Hes3*, its relevance to testosterone production remains unknown. To examine these matters, we performed *Mamld1* knockdown experiments.

**Methodology/Principal Findings:**

*Mamld1* knockdown was performed with two siRNAs, using mouse Leydig tumor cells (MLTCs). *Mamld1* knockdown did not influence the concentrations of pregnenolone and progesterone but significantly reduced those of 17-OH pregnenolone, 17-OH progesterone, dehydroepiandrosterone, androstenedione, and testosterone in the culture media. Furthermore, *Mamld1* knockdown significantly decreased *Cyp17a1* expression, but did not affect expressions of other genes involved in testosterone biosynthesis as well as in insulin-like 3 production. *Hes3* expression was not significantly altered. In addition, while 47 genes were significantly up-regulated (fold change >2.0×) and 38 genes were significantly down-regulated (fold change <0.5×), none of them was known to be involved in testosterone production. Cell proliferation analysis revealed no evidence for compromised proliferation of siRNA-transfected MLTCs.

**Conclusions/Significance:**

The results, in conjunction with the previous data, imply that *Mamld1* enhances *Cyp17a1* expression primarily in Leydig cells and permit to produce a sufficient amount of testosterone for male sex development, independently of the *Hes3*-related non-canonical Notch signaling.

## Introduction

*MAMLD1* (mastermind-like domain containing 1, alias *CXorf6*) on human chromosome Xq28 is a causative gene for hypospadias, a mild form of 46,XY disorders of sex development (DSD) [1]. To date, multiple mutations have been identified in patients with various types of hypospadias [1]–[3]. In this regard, the mouse homologous gene *Mamld1* is transiently expressed in fetal Sertoli and Leydig cells around the critical period for sex development [1], and transient *Mamld1* knockdown using small interfering RNAs (siRNAs) reduces testosterone (T) production in cultured mouse Leydig tumor cells (MLTCs) [4]. Furthermore, the upstream region of *MAMLD1*/*Mamld1* harbors a putative binding site “CCAAGGTCA” for *NR5A1* (alias, *SF-1* and *AD4BP*) [4] that regulates the transcription of a vast array of genes involved in sex development [5], and NR5A1 protein has been shown to bind to the putative target site and exert a transactivation function for *Mamld1* [4]. These findings imply that *MAMLD1*/*Mamld1* is involved in fetal T production under the regulation of NR5A1, and that *MAMLD1* mutations result in hypospadias primarily because of compromised T production around the critical period for sex development.

However, the underlying mechanism(s) by which impaired *MAMLD1*/*Mamld1* leads to compromised T production remains to be clarified, although there are several possibilities such as defective activities of enzyme(s) involved in T production and compromised proliferation of Leydig cells. Furthermore, although previous functional studies have indicated that *MAMLD1* has a transactivation function for the non-canonical Notch target *Hes3* [4], its relevance to biological function including T production remains unknown. To examine these matters, we performed detailed analyses in *Mamld1* knockdown experiments using MLTCs.

## Methods

### Knockdown experiments

MLTCs (ATCC, CRL-2065™) were maintained in RPMI 1640 supplemented with 10% fetal bovine serum, and were transiently transfected with two siRNAs, i.e., siRNA1 (sense: GCUUCCAGUUCAGAUGCCATT; and anti-sense: UGGCAUCUGAACUGGAAGCTT) and siRNA2 (sense: GGAACUAACCAAAAUUCAATT; and anti-sense: UUGAAUUUUGGUUAGUUCCTC) or with non-targeting control RNA (4611G) (final concentration 20 nM), using Lipofectamine RNAiMAX (Life Technologies). Relative amount of endogenous *Mamld1* mRNA against *B2m* (β2-microglobulin) was determined by the TaqMan real-time PCR method using the probe-primer mix on ABI PRISM 7000 (Life Technologies) (Assay No.: Mm01293665_m1 for *Mamld1*; and Mm00437762_m1 for *B2m*).

### Steroid metabolite measurements

MLTCs are known to have the capacity to produce T primarily via Δ^4^-pathway, although the amount of T production remains small primarily because of low 17α-hydroxylase and Hsd17b3 activities [6]. MLTCs are also known to retain responsiveness to human chorionic gonadotropin (hCG) [6]–[8]. Thus, after 48 hours of incubation of transfected MLTCs in 12-well plates with 1 ml of culture medium (an initial cell count: 1×10^5^ cells/well), hCG (Mochida Pharmaceutical) was added to the media at a final concentration of 50 IU/L, and the culture media were obtained at one hour after the addition of hCG. Subsequently, steroid metabolites in the T production pathway were measured by the liquid chromatography-tandem mass spectrometry (ASKA Pharma Medical). This experiment was performed three times.

### Gene expression analyses

Real-time reverse transcriptase (RT)-PCR and microarray analyses were performed using total RNA extracted from MLTCs that were harvested at the time of steroid metabolite measurements. For real-time RT-PCR analysis, 1 µg of total RNA was examined for relative mRNA dosage against *B2m* by the TaqMan Gene Expression Assay on ABI PRISM 7000 (Assay No.: Mm00446826_m l for *Nr5a1* (*Sf1*); Mm00441558_m1 for *Star*; Mm00490735_m1 for *Cyp11a1*; Mm01261921_mH for *Hsd3b1*; and Mm00484040_m1 for *Cyp17a1*). In addition to the genes for steroidogenic enzymes involved in T biosynthesis, we also studied *Insl3* (Mm01340353_m1) for gubernacular development that is expressed in Leydig cells [9], [10]. This experiment was repeated three times. For microarray analysis, 300 ng of total RNA was converted into cRNA associated with Cyanine-3 labeled CTP using RNA Spike-In Kit and Quick Amp Labeling Kit, and was subjected to hybridization on Whole Mouse Genome Oligo Microarray in triplicate (4×44 K G4122F) (Agilent Technologies). Subsequently, fluorescent signals were detected by Agilent Scanner, and were analyzed by GeneSpring GX10 (Tomy Digital Biology). The microarray data have been deposited in NCBI's Gene Expression Omnibus and are accessible through GEO series accession number GSE26913 (http://www.ncbi.nlm.nih.gov/geo/query/acc.cgi?acc=GSE26913). All data is MIAME compliant and the raw data has been deposited in a MIAME compliant database (GEO), as detailed on the MGED Society website (http://www.mged.org/Workgroups/MIAME/miame.html).

### Cell proliferation assays

The number of viable MLTCs transfected with two siRNAs or with non-targeting RNA was calculated by the colorimetric method [11], [12], using CellTiter 96 AQueous One Solution Cell Proliferation Assay (Promega). The detailed procedure has been described in the manufacturer's protocol. In brief, MLTCs were cultured in 96-well plates (an initial cell count: 1×10^4^ cells/well), and the cell number was determined every 24 hours by measuring the absorbance on a plate reader (Molecular Device) at 490 nm. This method is based on a positive correlation between the number of viable cells and the absorbance until the cells become confluent, and our preliminary studies showed a good correlation until the absorbance of ∼2.0 (∼6×10^5^ cells/well) (Figure S1). This experiment was performed three times.

### Statistical analysis

Statistical significance was examined by Student's *t*-test or by Mann-Whitney's *U*-test. *P*<0.05 was considered significant.

## Results

### Steroid metabolite measurements

**Figure 1:**
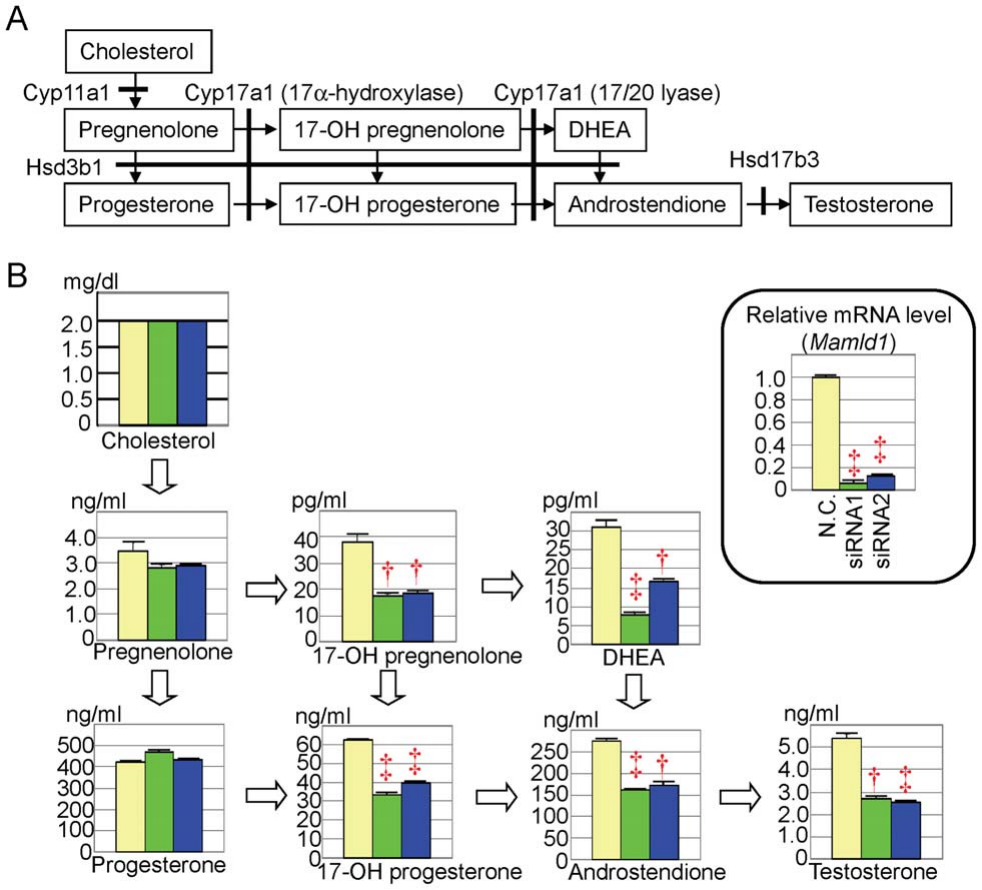
Steroid metabolite concentrations. A. Steroid metabolic pathway from cholesterol to testosterone and enzymes involved in each conversion. Pregnenolone, 17-OH pregnenolone, and DHEA (dehydroepiandrosterone) are Δ^4^-steroid metabolites (Δ^4^-pathway), and progesterone, 17-OH progesterone, and androstenedione are Δ^5^-steroid metabolites (Δ^5^-pathway). Hsd3b1 also functions as Δ^5 4^ isomerase. B. Steroid metabolite concentrations in culture media and endogenous *Mamld1* expression levels in MLTCs. The yellow, the green, and the blue bars indicate the data obtained from MLTCs transfected with non-targeting RNA, siRNA1, and siRNA2, respectively. †: *P*<0.01; and ‡: *P*<0.001. The conversion factor to the SI unit: cholesterol 0.026 (mmol/L), pregnenolone 3.16 (nmol/L), progesterone 3.18 (nmol/L), 17-OH pregnenolone 3.00 (pmol/L), 17-OH progesterone 3.03 (nmol/L), DHEA 3.46 (pmol/L), androstenedione 3.49 (nmol/L), and testosterone 3.46 (nmol/L).

The mean steroid metabolite concentrations are shown in Figure 1, together with the mean endogenous *Mamld1* mRNA levels that were markedly reduced in both siRNA1- and siRNA2-transfected MLTCs at the time of steroid metabolite measurements. The concentrations of pregnenolone and progesterone remained comparable between the culture media with siRNA-transfected MLTCs and those with non-targeted MLTCs, whereas the concentrations of 17-OH pregnenolone, 17-OH progesterone, dehydroepiandrosterone, androstenedione, and T were significantly lower in the culture media with siRNA-transfected MLTCs than in those with non-targeted MLTCs. Furthermore, comparison of the steroid metabolite concentrations in the media with non-targeted MLTCs confirmed revealed the Δ^4^-pathway dominant T production, markedly low 17α-hydroxylase activity and well preserved 17/20 lyase activity for both Δ^4^- and Δ^5^-pathways, and extremely low Hsd17b3 activity in MLTCs. These results indicated that *Mamld1* knockdown further reduced 17α-hydroxylase activity that was originally low in MLTCs.

### Gene expression analyses

**Figure 2:**
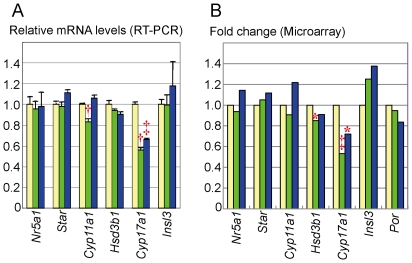
Gene expression analysis. The yellow, the green, and the blue bars indicate the data obtained from MLTCs transfected with non-targeting RNA, siRNA1, and siRNA2, respectively. *: *P*<0.05; †: *P*<0.01; and ‡: *P*<0.001. A. Real-time RT-PCR analysis. B. Microarray analysis.

Real-time RT-PCR and microarray analyses showed significantly decreased *Cyp17a1* expression (∼70%) in both siRNA1- and siRNA2-transfected MLTCs (Figure 2). Although *Cyp11a1* and *Hsd3b1* expressions were found to be reduced in siRNA1-transfected MLTCs by real-time RT-PCR and microarray analyses respectively, such reduced activities were not reproduced in siRNA2-transfected MLTCs. The siRNAs knockdown did not affect the expressions of *Nr5a1* (*Sf1*), *Star*, *Por*, and *Insl3*. The assessment of *Hsb17b3* was impossible, because of its extremely low expression.

In addition, 47 genes including a Notch-related gene *Hey1* were significantly up-regulated (fold change >2.0×) and 38 genes were significantly down-regulated (fold change <0.5×) in both siRNA1- and siRNA2-transfected MLTCs (Table S1 and Table S2). However, *Mamld1* knockdown had no discernible effect on the *Hes3* expression level (siRNA1: fold change 0.92, *P* = 0.80; siRNA2: fold change 1.43, *P* = 0.35). The microarray data have been deposited in NCBI's Gene Expression Omnibus and are accessible through GEO series accession number GSE26913 (http://www.ncbi.nlm.nih.gov/geo/query/acc.cgi?acc=GSE26913).

### Cell proliferation assays

**Figure 3:**
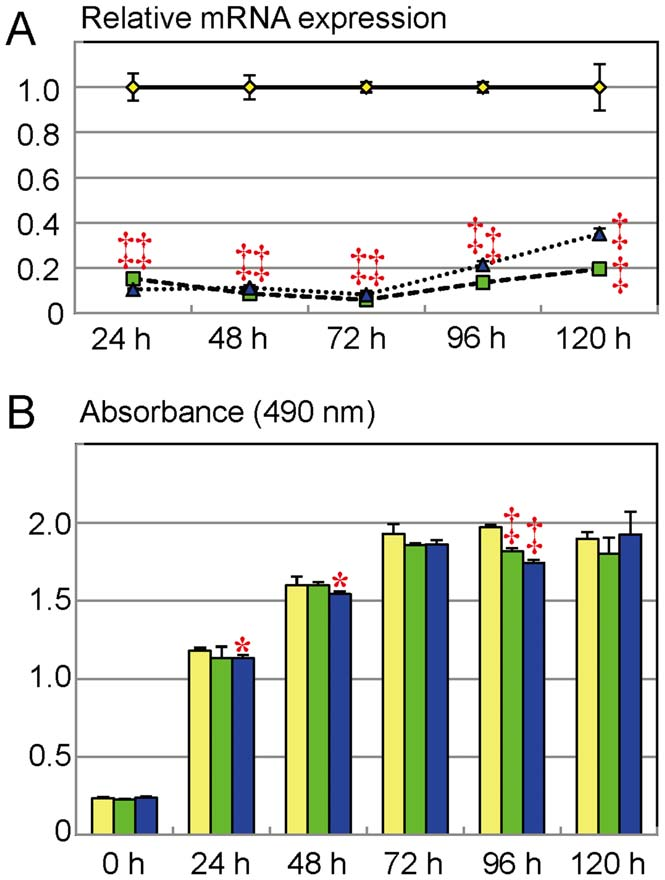
Cell proliferation assay. The yellow, the green, and the blue line graphs and bars indicate the data obtained from MLTCs transfected with non-targeting RNA, siRNA1, and siRNA2, respectively. *: *P*<0.05; and ‡: *P*<0.001. A. Endogenous *Mamld1* expression levels. B. Absorbance values.

The results are shown in Figure 3. The mean endogenous *Mamld1* mRNA levels were sufficiently suppressed for 120 hours in both siRNA1- and siRNA2-transfected MLTCs. Under this condition, the absorbance values for the siRNA-targeted and non-targeted MLTCs showed a roughly linear increase until 72 hours (absorbance ∼2.0). In this linear proliferative phase, although the absorbance values were significantly decreased in siRNA2-treated MLTCs at 24 and 48 hours after the transfection, this was not reproduced in siRNA1-transfected MLTCs. After 72 hours of incubation, the MLTCs became confluent, and the absorbance values became a plateau phase around ∼2.0. In this plateau phase, although the absorbance values at 96 hours after the transfection were significantly low in both siRNA1- and siRNA2-treated MLTCs, this was not reproduced at 120 hours after the transfection.

## Discussion

*Mamld1* knockdown with two siRNAs resulted in compromised T production, together with reduced 17α-hydroxylase activity and *Cyp17a1* expression in MLTCs. This provides further support for a positive role of *Mamld1* in T production [4], and implies for the first time a possible interaction between *Mamld1* and *Cyp17a1*, at least in MLTCs. In this regard, it is noteworthy that *Mamld1* is clearly expressed in fetal Leydig and Sertoli cells and is barely expressed in adrenal cells [1], [13], and that *Cyp17a1* expression is indispensable for T production in Leydig cells [14]. Thus, it appears likely that *Mamld1* enhances *Cyp17a1* expression primarily in Leydig cells, permitting the production of a sufficient amount of T for male sex development. In addition, since the expressions of other genes involved in T production and insulin-like 3 biosynthesis were not clearly affected in siRNA-transfected MLTCs, this would argue against the possibility that *Mamld1* knockdown causes a global dysfunction of MLTCs, resulting in T hyposecretion.

However, a straightforward explanation appears to be difficult between impaired 17α-hydroxylase activity and reduced *Cyp17a1* expression. Indeed, 17/20 lyase activity was well preserved in siRNA-transfected MLTCs, although the same Cyp17a1 enzyme is utilized for both17α-hydroxylase and 17/20 lyase reactions [14]. In addition, defective 17α-hydroxylase activity occurred in the presence of ∼70% of *Cyp17a1* expression, despite 17α-hydroxylase deficiency being an autosomal recessive disease in which 50% of enzyme reduction has no major effect on the steroid metabolism [14]. In this context, it is notable that MLTCs originally have a markedly low 17α-hydroxylase activity and a well preserved 17/20 lyase activity for both Δ^4^- and Δ^5^-pathways (Figure 1) [6]. Such a unique property of MLTCs may be relevant to the preferential impairment of 17α-hydroxylase activity in siRNA-transfected MLTCs.

*Mamld1* knockdown had no discernible effect on the *Hes3* expression. In addition, while a Notch-related gene *Hey1* [15]–[17] was up-regulated in siRNA-transfected MLTCs, there are no data suggesting a possible interaction between *Hes3* and *Hey1* in the T production process. Furthermore, while *Hes3* is weakly expressed in the MLTCs [4], *Hes3* expression is apparently absent from mouse fetal gonads around the critical period for sex development [18]. Thus, it is unlikely that *Hes3*-related non-canonical Notch signaling underlies a link between *Mamld1* and *Cyp17a1*. In addition, while microarray analysis revealed multiple up-regulated and down-regulated genes in siRNA-transfected MLTCs, none of them is known to be involved in the T production at present. It remains to be clarified, therefore, how *Mamld1* enhances *Cyp17a1* expression and T production.

The cell proliferation analysis revealed no clear evidence for the reduced number of viable MLTCs transfected with siRNAs. This implies that the reduced T and several other steroid metabolite concentrations observed at 48 hours after the transfection (Figure 1) are inexplicable by impaired proliferation of MLTCs. However, since the cell doubling time of MLTCs is 35–40 hours [8], a slight difference in cell proliferation would not be detected by the present analysis. Thus, it might remain tenable at this time that impaired cell proliferation becomes discernible after multiple cell divisions, and that such a possibly reduced cell proliferation underlies the development of hypospadias phenotype in patients with *MAMLD1* mutations, in addition to compromised T production in Leydig cells.

In summary, the present study implies that *Mamld1* enhances *Cyp17a1* expression primarily in Leydig cells and permit to produce a sufficient amount of T for male sex development, independently of the *Hes3*-related non-canonical Notch signaling. Although the data were obtained from *in vitro* studies using MLTCs, they provides a useful clue to clarify the underlying factors for the development of hypospadias and other forms of 46,XY DSD.

## Supporting Information

Figure S1 **Cell proliferation assay by the colorimetric method, using non-transfected MLTCs.** The absorbance value is well correlated with cell number until the absorbance value of ∼2.0, but does not reflect the cell number after the absorbance value of ∼2.0.
(TIF)

Table S1 List of up-regulated genes in MLTCs trasnfected with siRNAs for *Mamld1*.
(DOC)

Table S2 List of down-regulated genes in MLTCs trasnfected with siRNAs for *Mamld1*.
(DOC)
