# Supplementary figures and images for: Mamld1 Knockdown Reduces Testosterone Production and Cyp17a1 Expression in Mouse Leydig Tumor Cells
Source: PLoS One. 2011 Apr 29;6(4):e19123. doi: 10.1371/journal.pone.0019123 (PMC3084764; doi:10.1371/journal.pone.0019123)

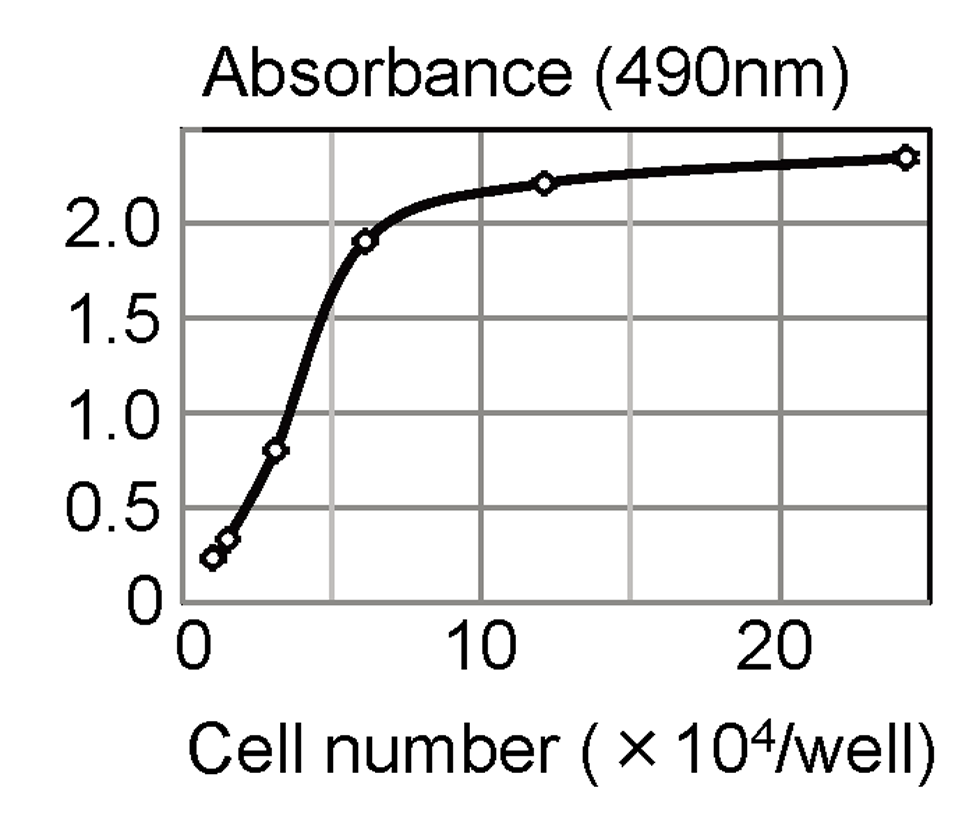

Supplement: Figure S1 — Cell proliferation assay by the colorimetric method, using non-transfected MLTCs. The absorbance value is well correlated with cell number until the absorbance value of ∼2.0, but does not reflect the cell number after the absorbance value of ∼2.0. (TIF) [file pone.0019123.s001.tif]
