# Supplementary material for: Mamld1 Knockdown Reduces Testosterone Production and Cyp17a1 Expression in Mouse Leydig Tumor Cells
Source: PLoS One. 2011 Apr 29;6(4):e19123. doi: 10.1371/journal.pone.0019123 (PMC3084764; doi:10.1371/journal.pone.0019123)
Supplement: Table S1 — List of up-regulated genes in MLTCs trasnfected with siRNAs for Mamld1. (DOC) [file pone.0019123.s002.doc]

| **Table S1.** List of up-regulated genes in MLTCs trasnfected with siRNAs for *Mamld1* | | | | | | |
| --- | --- | --- | --- | --- | --- | --- |
|  |  | siRNA1 | |  | siRNA2 | |
| Probe Name | Gene Symbol | Fold change | *P*-value |  | Fold change | *P*-value |
| A_52_P220695 | D030029J20Rik | 4.60 | 6.13E-06 |  | 6.26 | 5.74E-06 |
| A_52_P62833 | Foxred2 | 4.52 | 7.00E-05 |  | 6.07 | 1.60E-05 |
| A_52_P308448 | Vps53 | 5.43 | 5.91E-09 |  | 4.58 | 8.78E-07 |
| A_51_P350403 | Exoc4 | 3.91 | 1.79E-08 |  | 5.56 | 2.29E-07 |
| A_52_P956261 | AK086961 | 4.36 | 4.45E-05 |  | 4.05 | 9.83E-05 |
| A_52_P432289 | Asph | 3.68 | 1.51E-05 |  | 4.71 | 2.09E-05 |
| A_52_P373504 | Clasp2 | 3.27 | 3.27E-06 |  | 4.74 | 2.91E-06 |
| A_52_P156190 | Ednra | 5.34 | 3.04E-08 |  | 2.48 | 2.08E-05 |
| A_52_P62484 | D630042P16Rik | 4.68 | 5.01E-07 |  | 2.98 | 2.58E-05 |
| A_52_P493965 | Gucy1a3 | 3.64 | 3.71E-04 |  | 3.80 | 1.27E-03 |
| A_52_P148301 | Lysmd1 | 3.35 | 7.94E-08 |  | 3.20 | 1.70E-06 |
| A_51_P306527 | Ywhah | 3.09 | 1.90E-06 |  | 2.99 | 2.10E-05 |
| A_51_P389811 | Ccdc128 | 2.69 | 3.44E-05 |  | 3.35 | 1.82E-06 |
| A_52_P245277 | Spt1 | 2.88 | 1.22E-04 |  | 2.95 | 7.31E-04 |
| A_51_P202541 | Polr3e | 3.11 | 4.64E-07 |  | 2.56 | 1.21E-05 |
| A_52_P375003 | Blzf1 | 2.85 | 5.75E-04 |  | 2.82 | 2.33E-03 |
| A_51_P401894 | Nppc | 2.33 | 4.58E-04 |  | 3.24 | 7.99E-05 |
| A_51_P331279 | Arpc1b | 2.46 | 7.82E-05 |  | 3.03 | 8.06E-05 |
| A_52_P360595 | Eps15 | 2.79 | 5.73E-07 |  | 2.66 | 1.47E-06 |
| A_51_P516756 | Hipk3 | 3.02 | 1.43E-07 |  | 2.24 | 2.81E-05 |
| A_52_P401614 | D930048N14Rik | 2.71 | 1.53E-04 |  | 2.49 | 1.23E-03 |
| A_51_P102631 | Trappc2 | 2.49 | 5.05E-06 |  | 2.63 | 6.46E-05 |
| A_51_P408329 | AK089570 | 2.87 | 1.35E-04 |  | 2.21 | 1.57E-03 |
| A_51_P127334 | Zfp91 | 2.67 | 1.82E-04 |  | 2.33 | 1.20E-03 |
| A_52_P205603 | Olfr288 | 2.25 | 1.24E-04 |  | 2.69 | 6.98E-06 |
| A_51_P105927 | Rasl12 | 2.23 | 5.22E-05 |  | 2.68 | 1.38E-04 |
| A_52_P117576 | Casp3 | 2.27 | 3.25E-04 |  | 2.64 | 5.86E-04 |
| A_51_P476129 | Pwp2 | 2.37 | 2.12E-06 |  | 2.52 | 1.02E-05 |
| A_51_P292127 | Pgm2l1 | 2.48 | 2.60E-06 |  | 2.37 | 7.49E-06 |
| A_51_P456114 | 9930013L23Rik | 2.01 | 2.95E-03 |  | 2.80 | 1.95E-03 |
| A_51_P258409 | Hey1 | 2.14 | 1.54E-03 |  | 2.59 | 2.21E-03 |
| A_52_P197402 | 4930505D03Rik | 2.58 | 2.25E-06 |  | 2.12 | 6.02E-05 |
| A_51_P144264 | Klf2 | 2.05 | 9.93E-04 |  | 2.64 | 2.97E-04 |
| A_51_P213896 | Usp15 | 2.19 | 2.61E-06 |  | 2.49 | 1.47E-04 |
| A_52_P505944 | Samd8 | 2.25 | 4.43E-05 |  | 2.37 | 8.65E-04 |
| A_51_P266168 | Eif4e2 | 2.19 | 9.88E-06 |  | 2.29 | 1.21E-05 |
| A_51_P399217 | Pigx | 2.01 | 2.09E-07 |  | 2.43 | 4.01E-06 |
| A_51_P500544 | Mfsd1 | 2.29 | 2.86E-04 |  | 2.10 | 1.09E-05 |
| A_52_P300416 | Dcp1a | 2.31 | 2.36E-05 |  | 2.01 | 6.77E-05 |
| A_52_P26826 | Lage3 | 2.19 | 4.72E-05 |  | 2.13 | 2.85E-04 |
| A_51_P253348 | Chfr | 2.23 | 3.21E-05 |  | 2.06 | 3.68E-04 |
| A_52_P272945 | Ccnh | 2.20 | 2.87E-06 |  | 2.03 | 6.73E-05 |
| A_51_P202418 | Gopc | 2.11 | 2.86E-06 |  | 2.08 | 2.01E-06 |
| A_51_P463994 | A730008L03Rik | 2.08 | 5.85E-06 |  | 2.11 | 7.73E-05 |
| A_52_P512807 | Ccny | 2.02 | 9.50E-06 |  | 2.16 | 1.61E-05 |
| A_52_P463183 | Ddef1 | 2.11 | 2.98E-04 |  | 2.04 | 6.40E-04 |
| A_52_P384394 | Bmf | 2.08 | 3.20E-03 |  | 2.00 | 1.02E-04 |
