# Supplementary material for: Mamld1 Knockdown Reduces Testosterone Production and Cyp17a1 Expression in Mouse Leydig Tumor Cells
Source: PLoS One. 2011 Apr 29;6(4):e19123. doi: 10.1371/journal.pone.0019123 (PMC3084764; doi:10.1371/journal.pone.0019123)
Supplement: Table S2 — List of down-regulated genes in MLTCs trasnfected with siRNAs for Mamld1. (DOC) [file pone.0019123.s003.doc]

| **Table S2.** List of down-regulated genes in MLTCs trasnfected with siRNAs for *Mamld1ｓ* | | | | | | |
| --- | --- | --- | --- | --- | --- | --- |
|  |  | siRNA1 | |  | siRNA2 | |
| Probe Name | Gene Symbol | Fold change | *P*-value |  | Fold change | *P*-value |
| A_52_P590168 | Tg | 0.099 | 4.25E-06 |  | 0.159 | 1.03E-03 |
| A_51_P191865 | Lama2 | 0.121 | 1.94E-07 |  | 0.182 | 8.86E-09 |
| A_51_P428578 | 1810015C04Rik | 0.202 | 2.58E-03 |  | 0.208 | 1.04E-04 |
| A_51_P269546 | Trem2 | 0.329 | 7.34E-05 |  | 0.143 | 3.14E-05 |
| A_51_P512992 | Entpd3 | 0.315 | 1.96E-04 |  | 0.218 | 1.27E-04 |
| A_52_P319093 | Serpina3k | 0.247 | 3.34E-05 |  | 0.331 | 1.02E-03 |
| A_51_P510891 | Afp | 0.363 | 4.68E-04 |  | 0.256 | 2.73E-04 |
| A_52_P416046 | Dnali1 | 0.250 | 1.67E-05 |  | 0.408 | 2.91E-04 |
| A_52_P91043 | 5430420C16Rik | 0.356 | 4.11E-06 |  | 0.317 | 1.14E-04 |
| A_52_P488039 | NAP115231-1 | 0.255 | 2.86E-05 |  | 0.422 | 1.25E-03 |
| A_52_P522754 | Ebna1bp2 | 0.410 | 1.47E-04 |  | 0.289 | 1.29E-05 |
| A_51_P392740 | Spata18 | 0.396 | 4.05E-03 |  | 0.305 | 5.48E-06 |
| A_51_P282837 | St14 | 0.331 | 4.48E-05 |  | 0.371 | 1.45E-05 |
| A_51_P470460 | 4933405K07Rik | 0.351 | 3.31E-03 |  | 0.354 | 1.54E-03 |
| A_51_P501773 | Slc35f3 | 0.251 | 1.26E-07 |  | 0.463 | 3.77E-05 |
| A_52_P119350 | 4732419C18Rik | 0.381 | 1.07E-05 |  | 0.349 | 6.79E-05 |
| A_52_P286912 | NAP071160-1 | 0.317 | 3.36E-04 |  | 0.414 | 7.99E-04 |
| A_52_P326713 | Ccl25 | 0.394 | 1.73E-06 |  | 0.343 | 2.35E-06 |
| A_52_P63680 | Asah3 | 0.344 | 3.48E-06 |  | 0.405 | 3.58E-05 |
| A_51_P212191 | Txnl6 | 0.283 | 2.56E-06 |  | 0.474 | 3.60E-04 |
| A_52_P439001 | TC1648248 | 0.403 | 1.09E-07 |  | 0.361 | 5.23E-07 |
| A_51_P355996 | Acot5 | 0.453 | 7.58E-05 |  | 0.327 | 6.61E-05 |
| A_51_P199168 | Cidea | 0.478 | 1.19E-04 |  | 0.303 | 8.62E-05 |
| A_52_P507382 | Unc93b1 | 0.465 | 1.15E-04 |  | 0.319 | 1.40E-05 |
| A_52_P481316 | EG626359 | 0.371 | 1.60E-05 |  | 0.430 | 4.44E-04 |
| A_51_P451966 | Gml | 0.327 | 2.10E-05 |  | 0.480 | 1.11E-03 |
| A_52_P33831 | Lrrc27 | 0.375 | 1.18E-06 |  | 0.435 | 1.35E-04 |
| A_51_P483159 | Gchfr | 0.449 | 1.05E-04 |  | 0.371 | 1.86E-04 |
| A_51_P352216 | Zfp365 | 0.335 | 5.06E-04 |  | 0.494 | 3.31E-04 |
| A_51_P162162 | Inmt | 0.379 | 4.17E-06 |  | 0.464 | 9.92E-05 |
| A_51_P206824 | Hfe2 | 0.369 | 4.46E-06 |  | 0.489 | 9.27E-04 |
| A_51_P155323 | Hc | 0.463 | 7.85E-06 |  | 0.400 | 1.32E-05 |
| A_51_P177371 | Prnd | 0.489 | 1.75E-04 |  | 0.392 | 9.40E-05 |
| A_52_P426698 | Sema3b | 0.487 | 1.00E-04 |  | 0.394 | 2.18E-05 |
| A_52_P99517 | 4933434M16Rik | 0.425 | 8.28E-05 |  | 0.461 | 7.45E-04 |
| A_52_P299535 | AW548124 | 0.416 | 3.92E-05 |  | 0.492 | 8.98E-04 |
| A_51_P187726 | Dcun1d1 | 0.453 | 8.65E-04 |  | 0.463 | 1.13E-03 |
| A_52_P470150 | Ddc | 0.479 | 4.00E-04 |  | 0.461 | 3.24E-04 |
